# Factor affecting the discrepancy in the coronal alignment of the lower limb between the standing and supine radiographs

**DOI:** 10.1186/s12891-022-06099-7

**Published:** 2022-12-28

**Authors:** Hyun-Soo Moon, Sung-Hwan Kim, Dae-Kyung Kwak, Seung-Hun Lee, Yung-Hong Lee, Je-Hyun Yoo

**Affiliations:** 1 Arthroscopy and Joint Research Institute, Yonsei University College of Medicine, Seoul, Republic of Korea; 2 Department of Orthopedic Surgery, Hallym University Sacred Heart Hospital, Hallym University College of Medicine, Anyang, Republic of Korea; 3 Department of Orthopedic Surgery, Gangnam Severance Hospital, Yonsei University College of Medicine, Seoul, Republic of Korea

**Keywords:** Alignment discrepancy, Coronal alignment of the lower limb, Lower extremity, Full-length radiograph, Osteoarthritis, Body mass index

## Abstract

**Background:**

Conflicting results have been reported regarding the factors that can predict the discrepancy in the coronal alignment of the lower limb between radiographs taken in the standing and supine positions. Therefore, this study aimed to investigate factors that can predict discrepancies in the coronal alignment of the lower limb between radiographs taken in the standing and supine positions.

**Methods:**

We retrospectively evaluated the medical records of patients who underwent full-length anteroposterior radiographs of the lower limb in both standing and supine positions between January 2019 and September 2021. The discrepancy in the coronal alignment of the lower limb between the standing and supine radiographs was defined as the absolute value of the difference in the hip-knee-ankle (HKA) angle between the two radiographs, which is presented as the ΔHKA angle. Correlation and regression analyses were performed to analyse the relationship among ΔHKA angle, demographic data, and several radiographic parameters.

**Results:**

In total, 147 limbs (94 patients) were included in this study. The mean ΔHKA angle was 1.3 ± 1.1° (range, 0–6.5°). The ΔHKA angle was significantly correlated with body mass index and several radiographic parameters, including the HKA angle, joint line convergence angle, and osteoarthritis grade. Subsequent multiple linear regression analysis was performed using the radiographic parameters measured on the supine radiographs with the two separate models from the two observers, which revealed that body mass index and advanced osteoarthritis (Kellgren–Lawrence grades 3 and 4) had a positive correlation with the ΔHKA angle.

**Conclusions:**

Body mass index and advanced osteoarthritis affected the discrepancy in the coronal alignment of the lower limb between standing and supine radiographs. A discrepancy in the coronal alignment of the lower limb could be more prominent in patients with an increased body mass index and advanced osteoarthritis, corresponding to Kellgren-Lawrence grades 3 and 4.

## Background

Radiographic measurement of the coronal alignment of the lower limb is essential for establishing treatment strategies for knee diseases [1]. The treatment options for knee diseases, such as meniscal tears, articular cartilage lesions, and osteoarthritis, vary depending on the coronal alignment of the lower limb [2–6]. To plan a specific surgical procedure for a knee pathology, an evaluation of lower limb alignment should be performed (such as, high tibial osteotomy, and medial meniscus root repair). Furthermore, coronal alignment of the lower limb is used as a parameter to evaluate the clinical outcomes and prognosis after treatment (example, correction loss after high tibial osteotomy) [3, 7–10].

Most assessments of coronal alignment of the lower limb are performed with full-length radiographs taken while standing in weight-bearing status [11–14]. The knee joint consists of bony structures as well as soft tissues, such as ligaments, meniscus, and capsular structures, which could be affected by physiologic loading conditions [15]. Therefore, it is desirable to evaluate lower limb alignment in the standing position so that the actual physiologic load applied to the joint can be adequately reflected.

However, full-length radiographs of the lower limbs in the standing position are not available in all cases. For example, in patients with medial meniscus posterior root tears and advanced osteoarthritis, taking radiographs is often challenging because patients cannot bear full weight on the affected leg owing to pain. In such cases, an accurate assessment of lower-limb alignment with an actual loading condition cannot be made, which may affect the establishment of a treatment plan. In addition, intraoperative measurements of lower-limb alignment are inevitably performed in the supine position. Although measurements using radiographs taken in the supine position can be used, the measured values of coronal alignment of the lower limb may differ depending on the weight-bearing conditions [11, 14–19]. It has been reported that radiographs taken under weight-bearing conditions show a relatively higher degree of varus alignment than those taken under non-weight-bearing conditions [11, 15–17, 19, 20]. Therefore, information on factors that can predict the discrepancy in the coronal alignment of the lower limb between radiographs taken in the standing and supine positions is required, which has seldom been investigated. A few previous studies have investigated this topic, but related factors affecting the discrepancy in the coronal alignment of the lower limb were reported differently depending on the study (such as, age, body mass index, limb alignment, joint line convergence angle, and advanced osteoarthritis) [14, 20, 21]. Furthermore, these studies did not use a consistent imaging modality when measuring the lower limb alignment in the standing and supine conditions. Therefore, potential biases arising from the differences in imaging modalities should be considered. Accordingly, inconsistent findings among studies and their potential limitations suggest the need for further research. An analysis of this issue will provide knowledge on the relationship of the coronal alignment of the lower limb between radiographs taken in two different positions and may allow the prediction of lower limb alignment in a standing position using a radiograph taken in a supine position. This can help establish treatment strategies and evaluate clinical outcomes when full weight bearing is difficult or impossible in patients with severe pain due to knee disease.

Therefore, this study aimed to investigate factors that can predict discrepancies in the coronal alignment of the lower limb between radiographs taken in the standing and supine positions. Since body mass index is directly related to the mechanical load applied to weight-bearing joints [22], we hypothesized that it would influence the discrepancies in measurement results of lower limb alignment between radiographs taken in two different statuses.

## Methods

### Patient enrolment

**Fig. 1:**
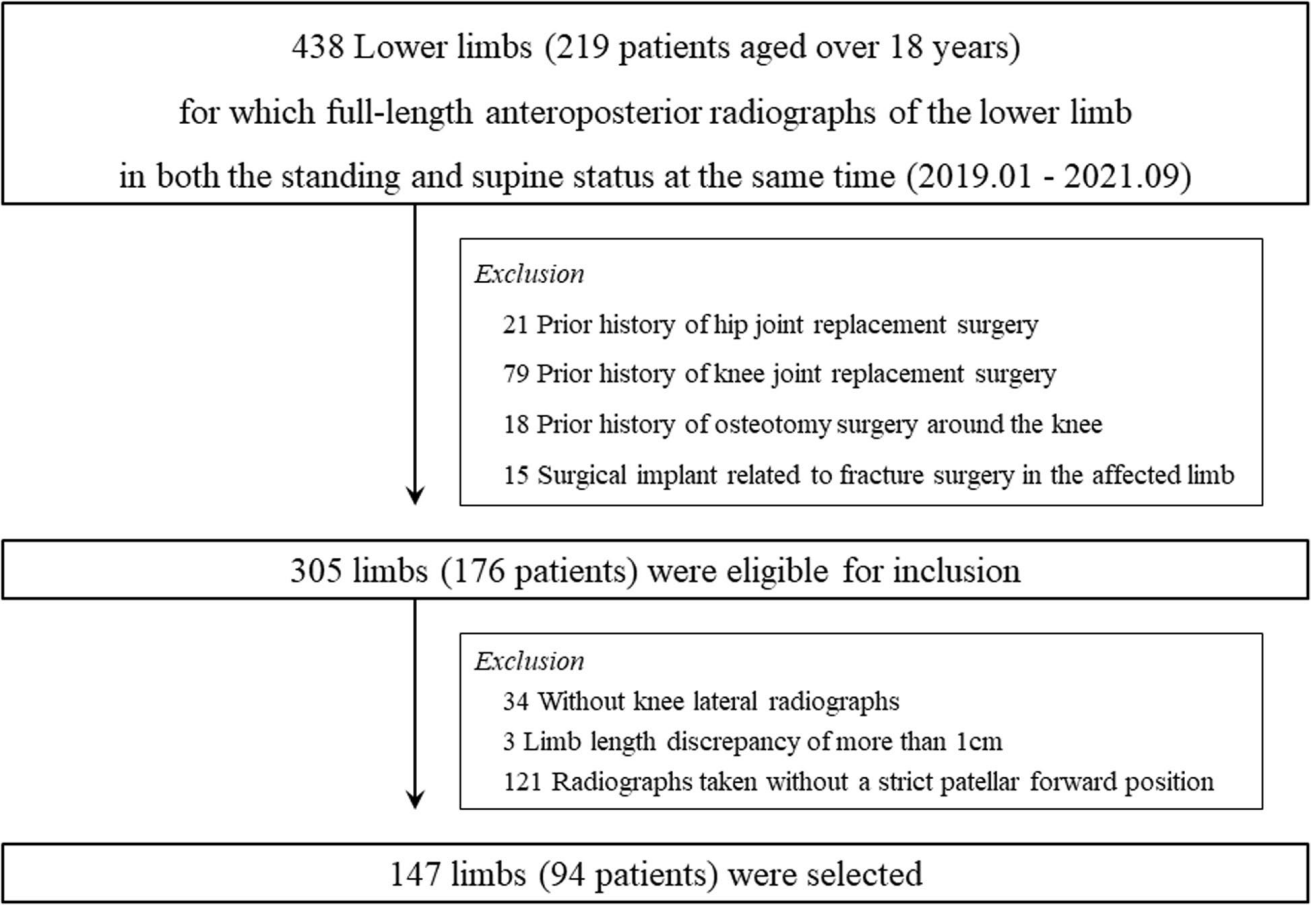
Flowchart of patient inclusion in the study

The present study was approved by the ethics committee of our institution, which waived the requirement for informed consent due to the retrospective nature of the study (IRB Number: 2022–05-015). The electronic medical records of patients who underwent full-length radiographs of the lower limbs at our institution between January 2019 and September 2021 were retrospectively reviewed. Patients aged > 18 years who underwent full-length anteroposterior radiographs of the lower limb in both standing and supine positions at the same time were included in the study. Of these, the lower limbs eligible for inclusion were included in this study. The exclusion criteria were as follows: a history of (1) hip joint replacement surgery; (2) knee joint replacement surgery; (3) osteotomy surgery; and (4) fracture surgery. In addition, (5) subjects without a lateral knee radiograph, (6) those with a limb length discrepancy of more than 1 cm, and (7) those with radiographs not taken in a strict patellar forward position in either standing or supine status were excluded (Fig. 1). Patients with a limb length discrepancy and full-length radiographs of the lower limb not taken in a strict patellar forward position were considered inadequate for analysis because knee joint rotation in the sagittal and axial planes could affect the accuracy of the coronal alignment measurement of the lower limb [23].

### Demographic data and image acquisition

For the baseline demographic data, age, sex, body mass index, side, and whether they corresponded to the affected limb were evaluated. Body mass index was calculated by dividing body weight in kilograms by height in meters squared. The affected limb represented the lower limb for which the patient complained of pain and was planned for evaluation or treatment.

All patients who were hospitalized in our institution with knee pain underwent full-length anteroposterior radiographs of the lower limb in both standing and supine positions. Standing radiographs were not taken for those who were unable to bear weight on the lower limb due to pain or discomfort. Images were taken with the patella facing towards the X-ray tube and at a focus-to-film distance of 300 cm (Innovision-SH, Shimadzu, Japan; GC85A, Samsung Electronics, Korea). The acquired images were stitched automatically into one composite image.

### Radiographic measurements

**Fig. 2:**
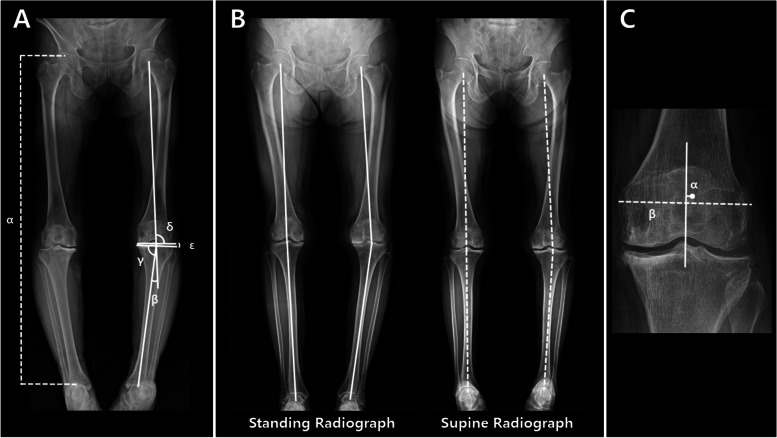
**A** Radiographic measurements of the length of the lower limb (α), HKA angle (β), medial proximal tibial angle (γ), lateral distal femoral angle (δ), and joint-line convergence angle (ε). **B** Full-length anteroposterior radiographs of the lower limb in both standing and supine position. The ΔHKA angle was defined as the absolute value of the difference between the acute angle generated by white solid lines and the acute angle formed by white dotted lines for each lower limb. **C** Patellar rotation was evaluated by calculating the degree of deviation of the patellar centre relative to the midpoint of the line connecting both femoral epicondyles (A/B * 100, %)

Radiographic parameters related to the coronal alignment of the lower limb were measured on both standing and supine radiographs. Radiographic variables included the hip-knee-ankle (HKA) angle, medial proximal tibial angle, lateral distal femoral angle, and joint line convergence angle (Fig. 2A) [24–27]. The discrepancy in the coronal alignment of the lower limb between the standing and supine radiographs was defined as the absolute value of the difference in the HKA angle between the two radiographs and represented the ΔHKA angle (Fig. 2B). The classification of coronal limb alignment was determined to be varus for HKA angle ≥2°, neutral from 2° to − 2°, and valgus for < − 2° based on the study by Moisio et al. [28]. To evaluate whether full-length anteroposterior radiographs of the lower limb were obtained in a patellar forward status, the position of the patella with respect to the femoral condyle was analysed (Fig. 2C) [23]. In this study, a patellar rotation of less than 3% was defined as a strict patellar forward position [23]. Limb length discrepancy was evaluated by comparing the length of both lower limbs according to the method published by Lang et al. [29] In addition, the radiographic osteoarthritis grade according to the Kellgren–Lawrence grading system was evaluated using radiographs of two different positions [30]. Assessments of the radiographic parameters on standing and supine radiographs were sequentially performed at an interval of 3 weeks to minimise bias. The posterior tibial slope was measured using the posterior tibial cortical line on lateral knee radiographs [31].

To increase the reliability of the outcome measures, all measurements were made by two orthopaedic surgeons who were blinded to patient information and each other’s findings using a picture archiving and communication system (INFINITT M6 6062 workstation, INFINITT Healthcare Co. Ltd., Korea). The average values of the measurements from the two observers were used for the analysis of continuous variables. Since categorical variables are qualitative variables and therefore cannot be present as average values, each measurement value from the two observers was used separately for the assessment of categorical variables.

### Statistical analysis

Statistical analyses were performed using IBM SPSS Statistics for Windows (v26.0; Armonk, NY, USA). A paired *t*-test was used to compare continuous variables measured on full-length radiographs of the lower limb taken in the standing position with those measured on radiographs taken in the supine position. McNemar–Bowker’s test was used to compare categorical variables. Pearson correlation and point-biserial correlation analyses were used to analyse the association between the ΔHKA angle and the other variables. Thereafter, multiple linear regression analyses with a stepwise method were conducted to identify the relationships and dependencies between the ΔHKA angle and the selected variables. Independent variables that were statistically significant in the preceding correlation analyses were eligible for inclusion in this model. In the regression model, categorical variables were converted into dummy variables to be analysed. Post hoc power for multiple linear regression analysis was calculated using G*POWER software (version 3.1.9.2; Heinrich Heine Universität, Düsseldorf) by setting the significance level at 5%. Inter-observer reliability was calculated using intra-class correlation coefficients set at a 95% confidence interval with a two-way random-effects model for continuous variables and a weighted kappa coefficient for categorical variables. The level of significance was set at *P* <  0.05.

## Results

In total, 147 limbs (94 patients) were included in this study. The baseline characteristics and radiographic data of the subjects are summarised in Table 1. The measurement reliability for radiographic parameters corresponding to continuous variables ranged from “good” to “excellent” (intra-class correlation coefficients, 0.861 to 0.994) [32]. For categorical variables, the measurement reliability was “substantial” (weighted kappa coefficients, 0.635–0.638) [33].

**Table 1:** Baseline demographic data and radiographic parameters

Variables^a^	Overall subjects (*n* = 147)^b^
Demographic data	
Age, year	64.6 ± 13.0
Sex	
Male/ Female	34/ 113
Body mass index, kg/m^2^	26.6 ± 4.3
Side	
Right/ Left	66/ 81
Affected limb	
Yes/ No	91/ 56
Radiographic parameters	
Full-length standing radiograph	
Hip-knee-ankle angle, °	5.2 ± 4.0
Limb alignment	
Varus/ Neutral/ Valgus	118/ 27/ 2
Medial proximal tibial angle, °	86.0 ± 2.1
Lateral distal femoral angle, °	88.1 ± 2.0
Joint line convergence angle, °	3.2 ± 2.4
Kellgren-Lawrence grade	
0/ 1/ 2/ 3/ 4 (observer 1)	44/ 37/ 23/ 25/ 18
0/ 1/ 2/ 3/ 4 (observer 2)	30/ 44/ 26/ 20/ 27
Full-length supine radiograph	
Hip-knee-ankle angle, °	4.3 ± 3.6
Limb alignment	
Varus/ Neutral/ Valgus	107/ 38/ 2
Medial proximal tibial angle, °	85.7 ± 2.3
Lateral distal femoral angle, °	88.3 ± 2.0
Joint line convergence angle, °	1.1 ± 1.9
Kellgren-Lawrence grade	
0/ 1/ 2/ 3/ 4 (observer 1)	43/ 40/ 28/ 23/ 13
0/ 1/ 2/ 3/ 4 (observer 2)	31/ 44/ 31/ 18/ 23
Knee lateral radiograph	
Posterior tibial slope, °	5.1 ± 2.4

^a^ The average value of the observers’ measurements was used for continuous variables, and each measurement value of observers was used separately for categorical variables

^b^ Data are presented as the mean ± standard deviation or number of limbs

The mean ΔHKA angle, which is the discrepancy in the coronal alignment of the lower limb between the standing and supine radiographs, was 1.3 ± 1.1° (range, 0°–6.5°). Pairwise comparisons between radiographic parameters measured on both the standing and supine radiographs showed statistically significant differences in most variables, except for the classification of limb alignment and medial proximal tibial angle (Table 2).

**Table 2:** Comparison of radiographic parameters measured on full-length radiographs of the lower limb taken in the standing and supine status

Variables^a^	Full-length standing radiograph^b^	Full-length supine radiograph^b^	*P* Value^c^
Hip-knee-ankle angle, °	5.2 ± 4.0	4.3 ± 3.6	< 0.001
Limb alignment			
Varus/ Neutral/ Valgus	118/ 27/ 2	107/ 38/ 2	0.056
Medial proximal tibial angle, °	86.0 ± 2.1	85.7 ± 2.3	0.083
Lateral distal femoral angle, °	88.1 ± 2.0	88.3 ± 2.0	< 0.001
Joint line convergence angle, °	3.2 ± 2.4	1.1 ± 1.9	< 0.001
Kellgren-Lawrence grade			
0/ 1/ 2/ 3/ 4 (observer 1)	44/ 37/ 23/ 25/ 18	43/ 40/ 28/ 23/ 13	0.007
0/ 1/ 2/ 3/ 4 (observer 2)	30/ 44/ 26/ 20/ 27	31/ 44/ 31/ 18/ 23	0.023

^a^ The average value of the observers’ measurements was used for continuous variables, and each measurement value of observers was used separately for categorical variables

^b^ Data are presented as the mean ± standard deviation or number of limbs

^c^ Paired *t* test was used for continuous variables, and McNemar-Bowker’s test was used for categorical variables

Correlation analysis was performed to evaluate the association between the ΔHKA angle and the other variables. The ΔHKA angle was significantly correlated with body mass index and several radiographic parameters, including the HKA angle, joint line convergence angle, and osteoarthritis grade (Table 3). Similar associations were observed in both cases when measured using supine and standing radiographs (Table 3).

**Table 3:** Correlation analysis between ΔHKA angle and variables

	*r* value	*P* Value^a^
ΔHKA angle and Demographic data		
Age	0.099	0.233
Sex	0.127	0.125
Body mass index	0.34	< 0.001
Side	0.017	0.841
Affected limb	− 0.077	0.357
ΔHKA angle and Radiographic parameters		
Full-length standing radiograph		
Hip-knee-ankle angle	0.467	< 0.001
Limb alignment	−0.057	0.055
Medial proximal tibial angle	−0.056	0.5
Lateral distal femoral angle	0.122	0.142
Joint line convergence angle	0.436	< 0.001
Kellgren-Lawrence grade (observer 1)	0.398	< 0.001
Kellgren-Lawrence grade (observer 2)	0.383	< 0.001
Full-length supine radiograph		
Hip-knee-ankle angle	0.205	< 0.013
Limb alignment	0.109	0.376
Medial proximal tibial angle	0.102	0.219
Lateral distal femoral angle	0.123	0.137
Joint line convergence angle	0.255	0.002
Kellgren-Lawrence grade (observer 1)	0.373	< 0.001
Kellgren-Lawrence grade (observer 2)	0.372	< 0.001
Knee lateral radiograph		
Posterior tibial slope	0.041	0.622

ΔHKA angle, the absolute value of the difference in the hip-knee-ankle angle measured in radiographs taken in the standing and supine status

^a^ Pearson correlation test was used for continuous variables, and Point-Biserial correlation test was used for categorical variables

**Fig. 3:**
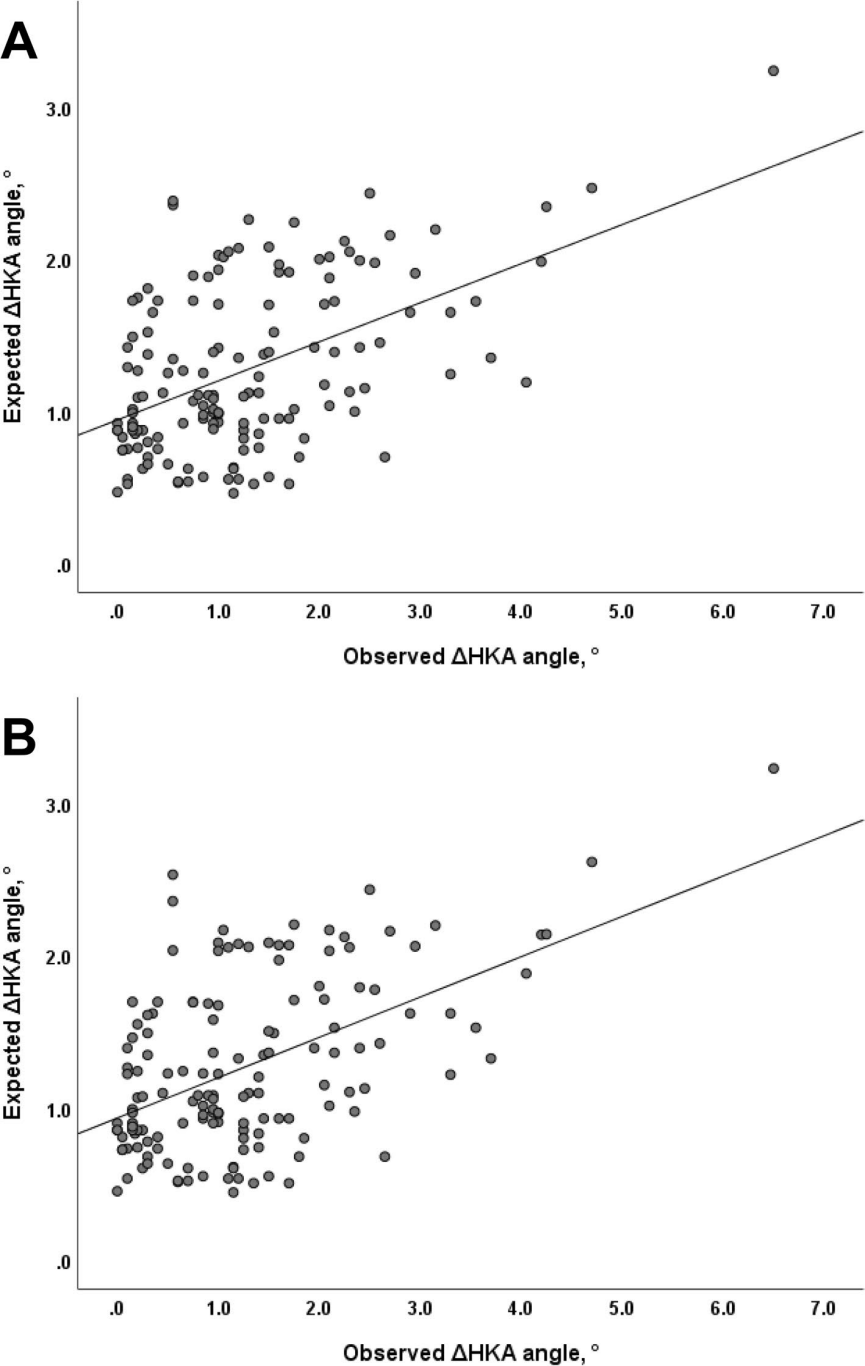
A linear correlation between the observed ΔHKA angle and the expected ΔHKA angle was noted in both the (A) Model 1 and (B) Model 2

Subsequent multiple linear regression analysis was performed to identify whether the correlated factors (body mass index, HKA angle, joint-line convergence angle, and osteoarthritis grade) independently affected the ΔHKA angle. Of the radiographic parameters eligible to be included in the regression model, only the variables measured using supine radiographs were entered into the model to predict lower limb alignment in a standing position using a radiograph taken in the supine position. In addition, since there were two measurement results for osteoarthritis grades, two separate regression models were used. As a result, body mass index and advanced osteoarthritis (Kellgren–Lawrence grades 3 and 4) were found to be independent factors that were positively correlated with the ΔHKA angle (Table 4). The corresponding findings were consistently observed in both models 1 and 2 (Model 1, Adjusted R^2^ = 0.241 and *P* <  0.001; Model 2, Adjusted R^2^ = 0.25 and *P* <  0.001) (Table 4). The regression models showed a linear correlation between the observed and expected ΔHKA angles (Fig. 3). The post-hoc power for the regression analysis in both models was more than 99.9%.

**Table 4:** Multiple linear stepwise regression analysis of the association between ΔHKA angle and selected variables

Variables	VIF	Beta	95% CI	*P* value
Model 1^a^				
Constant		−1.007	−1.979 to −0.036	0.042
Body mass index	1.012	0.077	0.04 to 0.113	< 0.001
Hip-knee-ankle angle (supine radiograph)	–	–	–	–
Joint line convergence angle (supine radiograph)	–	–	–	–
Kellgren-Lawrence grade (supine radiograph)				
1	–	–	–	–
2	–	–	–	–
3	1.02	0.894	0.464 to 1.323	< 0.001
4	1.03	1.043	0.491 to 1.595	< 0.001
Model 2^b^				
Constant		−1.007	−1.979 to −0.035	0.042
Body mass index	1.022	0.076	0.04 to 0.112	< 0.001
Hip-knee-ankle angle (supine radiograph)	–	–	–	–
Joint line convergence angle (supine radiograph)	–	–	–	–
Kellgren-Lawrence grade (supine radiograph)				
1	–	–	–	–
2	–	–	–	–
3	1.028	0.718	0.243 to 1.193	0.003
4	1.046	1.071	0.639 to 1.504	< 0.001

*VIF* Variance inflation factor, *CI* Confidence interval

^a^ Regression model based on the osteoarthritis grade measured by observer 1 (adjusted R^2^ = 0.241)

^b^ Regression model based on the osteoarthritis grade measured by observer 2 (adjusted R^2^ = 0.25)

## Discussion

The principal finding of this study was that a high body mass index and advanced osteoarthritis could affect the discrepancy in the coronal alignment of the lower limb between standing and supine radiographs. Body mass index and advanced osteoarthritis corresponding to Kellgren–Lawrence grades 3 and 4 showed a positive correlation with the ΔHKA angle, suggesting that a discrepancy in the coronal alignment of the lower limb between standing and supine radiographs could be more prominent in patients with increased body mass index and advanced osteoarthritis.

Full-length radiographs taken while standing are regarded as the gold standard modality for the assessment of the coronal alignment of the lower limb [11–14, 34]. Radiographs taken in the supine position can be used as an alternative, especially during surgery or when sufficient weight bearing is not possible. However, supine radiographs do not reflect physiologic loading conditions applied to the knee joint [15]. Also, it is well known that the coronal alignment of the lower limb can differ according to the weight-bearing condition [11, 14–19]. Therefore, for the evaluation of lower limb alignment using supine radiographs to have more clinical significance, information that can overcome its inherent limitations is required. If measurable factors affecting the discrepancies in the coronal alignment of the lower limb between radiographs taken in two different statuses can be identified, it may be possible to predict the lower limb alignment in a weight-bearing status using supine radiographs. When the lower limb alignment is measured on radiographs taken in the supine position and interpreted by considering related factors, a result close to the lower limb alignment measured on a standing radiograph can be inferred. Therefore, the relationship between the coronal alignment of the lower limb in standing and supine radiographs and the factors affecting the discrepancy thereof were analysed in the present study.

Similar to previous studies [11, 14–19], this study showed significant differences in pairwise comparisons of radiographic parameters regarding the coronal alignment of the lower limb measured in the standing and supine radiographs. The difference was not limited to the HKA angle but was also observed in most radiographic parameters, which are considered to have been influenced by the change in joint space according to weight-bearing conditions [15]. The load applied to the knee joint affects the soft tissue surrounding the knee joint, leading to a change in the joint space. This may have caused a difference in the overall measurement results of pairwise comparisons. The findings of this study support previous studies reporting that the measured values of radiographic parameters related to the coronal alignment of the lower limb may vary depending on weight-bearing conditions [11, 14–19].

Subsequently, correlation analysis and regression analysis were performed to identify variables affecting the discrepancy between the coronal alignment of the lower limb measured on radiographs taken in the standing and supine positions. Various factors, including age, body mass index, limb alignment, joint line convergence angle, and advanced osteoarthritis have been reported to influence the limb alignment discrepancy according to the weight-bearing status. However, this relationship had been shown inconsistently in previous studies [14, 20, 21]. The inconsistency in findings among these studies may be attributed to the following factors: First, each study had different patient characteristics (e.g., osteoarthritis grade) and variables for measurement [14, 20, 21]. Second, to evaluate the lower limb alignment in supine position, various tools such as magnetic resonance imaging, intraoperative fluoroscopy, and a navigation system have been used instead of simple radiographs in previous studies [14, 20, 21]. For a comprehensive assessment, this study included patients regardless of osteoarthritis grade, and various radiographic parameters were evaluated. In addition, the measurements of lower limb alignment in the standing and supine positions were equally conducted using plain radiographs. Hence, the bias resulting from the difference among the evaluation methods could be minimised. Moreover, the suitability of the images for measuring radiographic parameters was thoroughly checked by evaluating the limb length discrepancy and degree of patellar rotation during the subject selection process [23].

Consequently, this study revealed that in cases of increased body mass index and advanced osteoarthritis, the discrepancy in the coronal alignment of the lower limb between the standing and supine radiographs increased. It is well known that weight-bearing conditions can affect lower limb alignment [11, 14–19], and the results of this study coincide with this knowledge. An increased body mass index would increase the load applied to the knee joint [22], and advanced osteoarthritis would change the properties of the soft tissue surrounding the joint [35], which in turn affects lower limb alignment. It is important to note that the findings of this study are the results of the analysis, including most of the variables that affected the difference in the lower limb alignment according to weight-bearing in a strictly controlled condition. Accordingly, in patients with an increased body mass index and advanced osteoarthritis corresponding to Kellgren–Lawrence grade 3 or 4, special caution is required when evaluating coronal alignment of the lower limb using supine radiographs. When measuring coronal alignment of the lower limbs in patients with the corresponding factors, radiographs taken in the supine position may not be an appropriate alternative to standing radiographs. The predictive factors found in this study could be evidence-based parameters that should be considered when evaluating lower limb alignment when sufficient weight bearing is not possible or during surgery.

The present study had several limitations. First, this study was based on a retrospective review, which could be associated with a risk of bias in the evaluation. Second, there were very few cases of valgus alignment among the subjects included in this study. Therefore, the application of the findings of this study in patients with valgus alignment may be limited. Third, although this study found factors influencing the discrepancy between the coronal alignment of the lower limb measured on radiographs taken in the standing and supine positions, a specific cut-off value or equation could not be provided.

Full-length radiographs of the lower limbs taken in the supine position have been considered to have limited clinical availability owing to their potential limitations. Furthermore, even in the case of actual use, caution is required when interpreting the measurement results of lower-limb alignment. In this context, this study has strength in that it suggests a potential method to reduce limitations in interpreting results in supine radiographs by providing predictive factors that affect the discrepancy in the coronal alignment of the lower limb between standing and supine radiographs. If the predictive factors found in this study are sufficiently considered in the evaluation of the coronal alignment of the lower limb, the full-length radiograph of the lower limb taken in the supine position can be used as an appropriate alternative to standing radiographs, thereby providing a basis for expanding clinical usefulness.

## Conclusions

Body mass index and advanced osteoarthritis affected the discrepancy in the coronal alignment of the lower limb between standing and supine radiographs. A discrepancy in the coronal alignment of the lower limb could be more prominent in patients with an increased body mass index and advanced osteoarthritis, corresponding to Kellgren-Lawrence grades 3 and 4. Therefore, caution should be exercised when evaluating coronal alignment of the lower limb using supine radiographs in patients with these predictive factors.

## Data Availability

The datasets used and/or analyzed in this study available from the corresponding author on reasonable request.

## Abbreviations

HKA: Hip-Knee-Ankle
